# Evaluating the Integrated Management of Childhood Illness counselling skills of professional nurses in the North West Province of South Africa

**DOI:** 10.4102/hsag.v23i0.1074

**Published:** 2018-10-10

**Authors:** Marguerette-Francoisé Malan, Tinda Rabie, Catherina E. Muller

**Affiliations:** 1 School of Nursing Science NuMIQ Focus Area, North-West University, South Africa

## Abstract

**Background:**

The Integrated Management of Childhood Illness (IMCI) strategy provides guidelines for supporting and improving the health system to reduce under-5 children’s mortality rates. This strategy specifically assists professional nurses with the case management of children aged birth–5 years.

**Aim:**

The purpose of this study was to investigate how professional nurses provided counselling to caregivers of under-5 children based on the IMCI strategy in Primary Health Care facilities of one district in the North West Province of South Africa.

**Setting:**

Primary Health Care (PHC) facilities of one district in the North West Province.

**Method:**

A quantitative, descriptive and observational design was used. Counselling provided by the professional nurses was observed and a checklist was completed. This IMCI counselling checklist was based on aspects in the counselling section of the Health Facility Survey, formulated according to the IMCI strategy’s requirements.

**Results:**

Counselling that focused on feeding, administration of medication and counselling skills used during the consultation were good. However, counselling of caregivers of children aged 13 months to 5 years could be improved and the caregivers’ health status should also be addressed.

**Conclusion:**

Counselling provided to caregivers of under-5 children regarding feeding, administering of medication and caregivers’ health status used effective communication skills. However, technicalities of feeding such as lactation and nutritional guidance posed challenges.

## Introduction

Globally, under-5 child mortality continues to be a concern; even in developing countries, including South Africa, it remains a greater challenge despite the implementation of the Integrated Management of Childhood Illness (IMCI) strategy. The IMCI strategy was developed during the mid-1990s by the World Health Organization (WHO) and the United Nations International Children’s Emergency Fund (UNICEF) to address under-5 child mortality. This strategy provides guidelines for improving case management, assessment, provision of medication and counselling to reduce the under-5 child mortality rate (Chopra et al. 2005). South Africa was one of the 43 countries that adopted the IMCI strategy as the standard of care since 1997 to improve professional nurses’ skills and management of under-5 children’s illnesses, strengthening the health system support and improving family and community practices (Horwood et al. 2009; Victoria et al. 2006). This strategy aims to equip professional nurses with the necessary skills to classify, manage, refer and do follow-up of children as well counsel caregivers of under-5 children suffering from one or more illnesses (Victoria et al. 2006).

Despite the decline of under-5 child mortality in developed countries, challenges such as counselling and education, integrated planning and monitoring of data persist in developing countries (NDoH 2013). According to the United Nations (UN 2007), child mortality declined globally after the implementation of effective interventions such as the IMCI strategy. However, in sub-Saharan Africa (SSA), which includes South Africa, the incidence of under-5 mortality remains high, with an approximate reduction of 30% during 1990–2010 which is less than half of that initially aimed for by the Millennium Development Goals (MDGs), specifically MDG number 4 (Hill et al. 2012). According to Chopra et al. (2005), significant improvements occurred in the assessment of children after implementing IMCI, and the inappropriate usage of antibiotics and overall drug costs were reduced. However, the implementation of the IMCI strategy has its challenges, including evidence-based assessment, medication, effective and affordable usage of drugs, checking of immunisations and counselling of the caregivers. This lack of counselling could be attributed to time and human resource pressures encountered in South Africa’s public health care sector that serves 83% of the total South African population (Rabie, Klopper & Watson 2016:156) and because of nurses’ greater focus on assessment, examination and medication of the sick child than on counselling (Chopra et al. 2005). Professional nurses failed to ask for the children’s Road to Health Booklets (RtHBs), which resulted in failure to plot weights, and thus failure to assess under-5 children. This implies that potentially inadequate counselling was provided by professional nurses to caregivers regarding the appropriate return dates to health facilities (Chopra et al. 2005; Tarwa & De Villiers 2007). Therefore, this study specifically focused on the counselling provided to caregivers of under-5 children, including the use of medications at home, non-medicinal health education based on the illness, return dates, nutrition and feeding advice.

According to Liu et al. (2012), 7.6 million under-5 children globally died in 2010, of whom 4.8 million died from infectious diseases such as pneumonia and diarrhoea. Statistics South Africa (2014) indicated that the North West Province and Northern Cape Province of South Africa reported 7.5% under-5 death rates, which were the highest in South Africa. In the North West Province, Ngaka Modiri Molema District indicated the highest under-5 mortality rate of 137.3 deaths per 1000 live births during 2009 (South Africa National Department of Health [NDoH] 2012). Initiatives have been implemented by the South African government to limit under-5 child mortality rates because from 1998 to 2007 South Africa experienced an increase in under-5 deaths from 38 to 67 per 1000 live births (NDoH 2013). According to the WHO (2014), this number was higher than South Africa’s average figures with 78 under-5 deaths per 1000 live births. The leading causes of death included diarrhoea, HIV and/or AIDS, malnutrition, other infectious diseases and acute respiratory diseases such as pneumonia, all of which are addressed by the IMCI strategy. All nine provinces of South Africa showed a decline in under-5 child mortality, with the greatest decline in the North West Province from 2007 to 2009. This decline could be ascribed to improved prevention and management of diseases through the implementation of the IMCI strategy (NDoH 2012).

### Research problem statement

The literature shows that under-5 mortality remains a problem in South Africa despite interventions implemented to reduce it. Effective preventative measures were implemented through the IMCI strategy to address the under-5 mortality rate. According to Krug and Pattinson (2004), a study undertaken in Mafikeng indicated a lack of proper communication (including counselling) between the professional nurses and caregivers, resulting in failure to implement the IMCI strategy effectively.

### Purpose (aims) and objectives

The purpose of this study was to investigate how professional nurses provided counselling to caregivers of under-5 children based on the IMCI strategy at Primary Health Care (PHC) facilities in the North West Province. The objective was to describe how IMCI counselling was conducted in PHC facilities of one district of the North West Province.

### Definitions of key concepts

*Caregiver* refers to any person providing mothering activities to an under-5 child (Madau 2010:5).

*Counselling* is an art based on personality, values, skill and providing the necessary knowledge (Okun & Kantrowitz 2007:13).

*Integrated Management of Childhood Illness strategy* focuses on set guidelines to improve case management, assessment, provision of medication and counselling to reduce under-5 child mortality rates (Atun et al. 2010; Chopra et al. 2005).

*Primary Health Care facility* is a primary health service level where preventative, promotional and curative services are provided to the community.

*Under-5 mortality rate* describes the deaths among children from birth to 5 years of age, divided by the numerical value of live births and articulated as the rate per 1000, in an under-5 year population of a particular year (Malishane 2012).

## Materials and methods

### Design

A quantitative, observational and descriptive design was used.

### Population, sampling and sample size

The population of this study consisted of IMCI-trained professional nurses (*N* = 49; *n* = 23) providing counselling to caregivers of under-5 children during consultations in 10 (*n* = 10) PHC facilities of one district in the North West Province. Although the population of IMCI-trained professional nurses in the district was 49, only 23 professional nurses’ counselling skills were observed as some were off duty, went for training or worked night shifts. A total of 237 consultations were observed.

### Data collection procedures

Data were collected by using a checklist based on the IMCI counselling section of the Health Facility Survey grounded on the IMCI strategy. The Health Facility Survey was developed by the Ministry of Health and Population, Egypt, and the WHO Regional Office for the Eastern Mediterranean countries (WHO 2003). Before data collection, a statistician assisted in adapting the checklist by changing the Yes/No question format of the checklist to a six-point Likert scale, which included not at all, very little, fairly well, quite well, very well, perfectly and not applicable. The questions were based on the following aspects.

*Feeding counselling (Items A1–A14 and A16)*: A1 – Does the professional nurse explain the importance of breast or formula feeding? A2 – Does the professional nurse explain the frequency of breast or formula feeding? A3 – Does the professional nurse explain how to improve lactation? A4 – Does the professional nurse identify the mother’s concerns and manage any breast or formula feeding problems? A5 – Does the professional nurse give guidance regarding correct positioning and attachment during breast or bottle feeding? A6 – Does the professional nurse provide the mother with advice when breast or formula feeding is discontinued? A7 – Does the professional nurse ensure that additional or substitute milk is prescribed according to the nutritional guidelines? A8 – Does the professional nurse give information regarding the hygienic preparation of formula milk before feeding? A9 – Does the professional nurse advise the caregiver regarding the length of time that breast or formula milk can be stored? A10 – Does the professional nurse show how to feed with a cup for babies from 6 months and older? A11 – Does the professional nurse advise on complementary foods and frequency of feeding? A12 – Does the professional nurse give advice on alternatives when the child refuses all milk? A13 – Does the professional nurse give counselling regarding nutritional feeding? A14 – Does the professional nurse give information regarding continuation of milk or breastfeeding after nutritional meals? and A16 – Does the professional nurse review the RtHB and give counselling to the caregiver?

*Feeding counselling of the child between 13 months and 5 years (Items A15.1–A15.2)*: A15.1 – Keeping record of what the child eats. A15.2 – Serving finger foods as often as possible. A15.3 – Introduction of new food – one at a time together with familiar food. A15.4 – Do not pressure the child to eat all the food on the plate. A15.5 – Give the child a choice of food, containing similar nutrients.

*Administration of medication (Items B1–B8)*: B1 – Does the professional nurse explain what the oral treatment is for? B2 – Does the professional nurse explain how to administer the oral treatment? B3 – Does the professional nurse demonstrate how to administer the oral treatment? B4 – Does the professional nurse ask open-ended questions to evaluate if the caregiver understands how to administer the oral treatment? B5 – Does the professional nurse demonstrate how to administer the first dose of the oral drug to the child under-5 at the facility? B6 – Does the professional nurse advise and explain when the caregiver should return to the clinic for a follow-up visit? B7 – Does the professional nurse prescribe or give oral rehydration solution treatment? B8 – Does the professional nurse explain how to mix and administer oral rehydration solution correctly?

*Consideration of caregivers’ health during counselling (Items C1–C3)*: C1 – Does the professional nurse inquire if the caregiver is healthy? C2 – Does the professional nurse advise the caregiver to eat well, to build his or her own strength? C3 – Does the professional nurse encourage the caregiver to talk about social difficulties he or she may experience?

*Counselling skills during consultation (Items D1–D3, D5 and D7)*: D1 – Active listening. D2 – Inviting questions. D3 – Praising the caregiver. D5 – Showing interest in the caregiver. D7 – Evaluating the caregivers’ understanding.

*Use of simple language, kindness and respect towards the caregiver (Items D4 and D6)*: D4 – Using simple language. D6 – Talking kindly and respectfully.

During data collection, the data collector observed the entire consultation process of the professional nurses’ counselling given to the caregiver of the under-5 child and ticked the checklist. The data collector was alone with the professional nurse, caregiver and under-5 child in the consultation room. Informed consent was obtained from both the professional nurse and the caregiver of the under-5 child. Before the consultation took place, the aim of the research study was explained to the caretaker, then verbal approval was obtained and finally the caretaker signed an informed consent form. The duration of the professional nurses’ consultations ranged between 10 and 60 min, depending on the illness(es) of the particular under-5 child.

### Data analysis

Data analysis included descriptive statistics and exploratory factor statistics.

#### Descriptive statistics

Descriptive statistics included feeding counselling (A1–A14 & A16) (see Table 1), administration of medication (B1–B8) (see Table 2) and nurses’ use of simple language, kindness and respect towards the caregiver (D4 and D6) (Table 3). The description designated the number of observed IMCI counselling sessions. The mean score and standard deviation were reported. If the mean was 2.5 and above for a certain item, it means that item was present during counselling; and if the mean score was below 2.5, it means that particular item was absent during counselling.

**TABLE 1:** Feeding counselling.

Items	Description of items	*M*	SD
A1	Does the professional nurse explain the importance of breast or formula feeding?	4.73	0.70
A2	Does the professional nurse explain the frequency of breast or formula feeding?	3.50	1.52
A3	Does the professional nurse explain how to improve lactation?	1.35	1.61
A4	Does the professional nurse identify the mother’s concerns and manage any breast or formula feeding problems?	0.76	1.60
A5	Does the professional nurse give guidance regarding correct positioning and attachment during breast or bottle feeding?	3.79	1.95
A6	Does the professional nurse provide the mother with advice when breast or formula feeding is discontinued?	2.63	2.25
A7	Does the professional nurse ensure that additional or substitute milk is prescribed according to the nutritional guidelines?	2.03	2.21
A8	Does the professional nurse gives information regarding the hygienic preparation of formula milk before a feeding?	3.23	1.80
A9	Does the professional nurse advise the caregiver regarding the length of time that breast or formula milk can be stored?	1.71	2.14
A10	Does the professional nurse show how to feed with a cup for babies from 6 months and older?	3.46	2.13
A11	Does the professional nurse advise on complementary foods and frequency of feeding?	2.61	2.06
A12	Does the professional nurse give advice on alternatives when the child refuses all milk?	0.88	1.64
A13	Does the professional nurse give counselling regarding nutritional feeding?	1.87	2.05
A14	Does the professional nurse give information regarding continuation of milk or breastfeeding after nutritional meals?	2.68	2.23
A16	Does the professional nurse review the RtHB and give counselling to the caregiver?	4.98	0.33

RtHB, Road to Health Booklet; *M*, mean; SD, standard deviation.

**TABLE 2:** Administration of medication.

Items	Description of items	*M*	SD
B1	Does the professional nurse explain what the oral treatment is for?	4.88	0.34
B2	Does the professional nurse explain how to administer the oral treatment?	4.90	4.91
B3	Does the professional nurse demonstrate how to administer the oral treatment?	3.09	2.31
B4	Does the professional nurse ask open-ended questions to evaluate if the caregiver understands how to administer the oral treatment?	2.95	2.44
B5	Does the professional nurse demonstrate how to administer the first dose of the oral drug to the child under-5 at the facility?	2.68	2.68
B6	Does the professional nurse advise and explain when the caregiver should return to the clinic for a follow-up visit?	4.59	1.14
B7	Does the professional nurse prescribe or give oral rehydration solution for treatment?	5.00	0
B8	Does the professional nurse explain how to mix and administer oral rehydration solution correctly?	5.00	0

*M*, mean; SD, standard deviation.

**TABLE 3:** Use of simple language, kindness and respect towards the caregiver.

Items	Description of items	*M*	SD
D4	Using simple language	4.36	0.75
D6	Talking kindly and respectfully	4.05	0.48

*M*, mean; SD, standard deviation.

#### Exploratory factor analysis

Exploratory factor analysis assisted to analyse data in sets in which their descriptions were facilitated. Exploratory factor analysis could only be used on certain data, as not all the IMCI counselling checklist’s items (see Table 4) were applicable to all children, as certain items focused on different age groups. Data sets were analysed using the Statistical Analysis System (SAS) version 9.3 standardised program. During data analysis, a mean score was determined by giving each item a score to evaluate the counselling provided by each professional nurse. A score of 0 was given when no counselling was given, a score of 1 when very little counselling was given, a score of 2 when counselling was fairly well done, a score of 3 for counselling done quite well, a score of 4 for counselling given very well and a score of 5 when counselling was perfectly done. There were initially four different factors: feeding counselling of a child between 13 months and 5 years (Factor 1; Items A15.1–15.5), consideration of the caregivers’ health during counselling (Factor 2; Items C1–C3), use of counselling skills during consultations (Factor 3; Items D1–D3, D5 and D7); however Items D4 and D6 referring to simple language, kindness and respect towards the caregiver, which initially comprised part of Factor 3 split during analysis and, therefore, these two items were excluded and descriptive statistics were used for those two items because of their unacceptable Cronbach’s alpha coefficients (see Table 3).

**TABLE 4:** Exploratory factor analysis.

Factors	Item numbers	Items	*M*	SD	Cronbach’s alpha
Factor 1 Feeding counselling of the child between 13 months and 5 years	A15.1	Keeping record of what the child eats	2.18	1.60	0.61
	A15.2	Serving finger foods as often as possible			
	A15.3	Introduction of new food – one at a time together with familiar food			
	A15.4	Do not pressure the child to eat all the food on the plate			
	A15.5	Give the child a choice of food, containing similar nutrients			
Factor 2 Consideration of caregivers’ health during counselling	C1	Does the professional nurse inquire if the caregiver is healthy?	0.48	0.97	0.69
	C2	Does the professional nurse advise the caregiver to eat well, to build his or her own strength?			
	C3	Does the professional nurse encourage the caregiver to talk about social difficulties he or she may experience?			
Factor 3 Counselling skills during consultation	D1	Active listening	2.78	0.79	0.59
	D2	Inviting questions			
	D3	Praising the caregiver			
	D5	Showing interest in the caregiver			
	D7	Evaluating the caregivers’ understanding			

*M*, mean; SD, standard deviation.

### Ethical considerations

After permission was granted by the PHC facility, professional nurse and caregiver of the under-5 child, data collection commenced. Counselling given by the professional nurses during the consultation of the under-5 children accompanied by their caregivers was observed. Data collection could be terminated if this was requested by the professional nurse or caregiver. However, there were no such requests. The researcher respected the professional nurses’ professional knowledge and vulnerability of the caregivers and the under-5 children. The professional nurse and caregiver signed voluntary informed consent forms to permit the researcher to be present during the IMCI case management practice. No personal information which could identify the PHC facility, professional nurse providing the counselling, under-5 child or caregiver receiving the counselling was collected; only codes were used on the checklists. No information collected during the observations of IMCI counselling sessions was shared with others. The researcher ensured that no professional nurse or caregiver was exploited and that all checklists were handled confidentially.

Ethical clearance was obtained from the Health Research Ethics Committee of the Faculty of Health Sciences of the North-West University (NWU), Potchefstroom Campus (NWU-00079-15-A1). Written approval was obtained from the North West Provincial Department to conduct the study.

### Reliability and validity

The confirmatory factor analysis confirms the validity of the IMCI counselling observations for this study. Statistics for this study focused on the counselling provided by the professional nurses to the caregivers. Cronbach’s alpha coefficients were reliable and ranged between 0.59 and 0.69. Factor 1, feeding counselling of the child between 13 months and 5 years, had a Cronbach’s alpha coefficient of 0.61; Factor 2, consideration of caregivers’ health during counselling, had a Cronbach’s alpha coefficient of 0.69; and lastly Factor 3, the use of counselling skills during consultations, had a Cronbach’s alpha coefficient of 0.59. All three factors were accepted as being reliable.

## Results and discussion

### Descriptive statistics

Table 1 (A1–A14 & A16) addresses feeding counselling regarding good practices in breast and/or formula feeding and challenges experienced. Professional nurses explained the importance of breastfeeding or formula feeding to the caregivers (mean = 4.73), including the frequency of breast and formula feeding (mean = 3.50). Professional nurses did not counsel on ways to improve lactation (mean = 1.35) nor did they identify the mothers’ concerns to manage any breast or formula feeding problems (mean = 0.76). The professional nurses counselled the caregivers on positioning of the baby and attachment during breast or bottle feeding (mean = 3.79). Professional nurses counselled caregivers about the expected time when to discontinue breast and bottle feeding (mean = 2.63), but did not prescribe additional or substitute milk according to the nutritional guidelines (mean = 2.03). There was a lack of information regarding hygienic preparation of formula milk before feeding (mean = 3.23). Information on the length of time breast or formula milk could be stored (mean = 1.71) was poor. Most caregivers were shown how to cup feed a baby from the age of 6 months (mean = 3.46). Some professional nurses counselled the caregivers about complementary feeding (mean = 2.61) but did not advise them about alternative foods if the child refused milk (mean = 0.88). Nutritional counselling on feeding was not good (mean = 1.87), but counselling on continuation of milk or breastfeeding after nutritional meals (mean = 2.68) was good. Almost all professional nurses reviewed the RtHBs and counselled the caregivers appropriately (mean = 4.98). This finding of the study seemed to contradict Chopra et al.’s (2005) report that professional nurses failed to request the children’s RtHBs. This improvement might be attributed to the implementation of the IMCI strategy and to the training of professional nurses in this strategy.

Table 2 (Items B1–B8) focuses on the administration of medication. Items focusing on whether the professional nurse explained what oral treatment was required for (mean = 4.88) and how to administer the oral treatment (mean 4.90) both had excellent mean scores. Demonstrations of how to administer oral treatment (mean = 3.09) were good and the professional nurses asked open-ended questions to evaluate whether the caregivers understood how to administer oral treatment (mean = 2.95). Demonstrations of giving the first dose of oral drugs to children (mean = 2.68) were good. Most professional nurses advised and explained when the caregivers should return to the clinics for a follow-up visit (mean = 4.59). Nurses’ prescriptions for oral rehydration solution (mean = 5.00), and explaining how to mix and administer this solution (mean = 5.00), had perfect mean scores.

The additional items (Items D4 and D6), which focused on the use of simple language and kindness and respect towards the caregivers which were excluded from the factor counselling skills during consultation, were independently analysed under descriptive statistics. Item D4 indicated that most professional nurses used simple language (mean = 4.36) and Item D6 indicated that the professional nurses talked kindly and respectfully (mean 4.05) during counselling (see Table 3).

### Exploratory factor statistics

The exploratory factor analysis indicated that Factor 1 (Items A15.1-A15.5), namely the feeding counselling of the child between 13 months and 5 years (mean = 2.18), was present. The score for Factor 2 (Items C1-C3), consideration of caregivers’ health during counselling (mean = 0.48), was lower than 2.5, indicating that these aspects were not evident during the caregivers’ counselling sessions. Factor 3 (Items D1-3, D5 & D7), addressing counselling skills during consultation (mean = 2.78), obtained a mean score of above 2.5, indicating that effective counselling skills were observed during the consultations (see Table 4).

### Limitations

The first limitation of this study was that some PHC facilities did not have the latest version of the IMCI booklet. Secondly, the Hawthorne effect could have been possible because the professional nurses knew their consultations were being assessed by the researcher and lastly not all IMCI-trained professional nurses could participate in this study as some were on leave or working night shifts.

### Recommendations for future research

Future research should be conducted on the counselling skills of professional nurses who did not do the IMCI course but who worked in PHC facilities as well as evaluation of nursing graduates’ counselling skills after completing the IMCI course.

## Conclusions

The descriptive statistics on the IMCI counselling provided to the caregivers of under-5 children included feeding counselling, administration of medication and the use of simple language and displaying kindness and respect towards the caregivers. Professional nurses explained the importance of breastfeeding, but certain information related to breastfeeding, such as lactation, feeding concerns of the caregivers, storage of breast and formula milk and alternative feeding when a child refuses all milk, required more attention. Information regarding additional or substitute milk was not provided according to the nutritional guidelines. The results regarding the administration of medication were relatively good, but attention could be given to the evaluation of the caregivers’ understanding of how to administer the first dose oral medicines.

The exploratory factor analysis of Factor 1, feeding counselling of the child between 13 months and 5 years, indicated that the introduction of new foods in this age group posed challenges. Factor 2 indicated that caregivers’ health was not considered during counselling, requiring much more attention. The last factor (Factor 3) indicated that the counselling skills implemented by the professional nurses during the consultation of caregivers of the under-5 children were relatively good.
